# Blood-brain barrier dysfunction assessed by protein S-100 beta levels in cirrhotic patients in ICU

**DOI:** 10.1186/2197-425X-3-S1-A813

**Published:** 2015-10-01

**Authors:** N Weiss, D Monneret, F Imbert-Bismuth, S Tripon, M Mallet, M Rudler, D Thabut, Brain Liver Pitie-Salpetriere Study Group (BLIPS)

**Affiliations:** Assistance Publique - Hopitaux de Paris, La Pitié-Salpetriere Hospital, Neurological ICU, Neurology Department, Paris, France; Assistance Publique - Hopitaux de Paris, La Pitié-Salpetriere Hospital, Biochemestry Department, Paris, France; Assistance Publique - Hopitaux de Paris, La Pitié-Salpetriere Hospital, Paris, France; Assistance Publique - Hopitaux de Paris, La Pitié-Salpetriere Hospital, Hepatological ICU, Paris, France

## Introduction

The protein S-100 beta (PS-100) is a small dimeric cytosolic protein synthetized in astrocytes and Schwann cells. High serum levels of PS-100 are associated with brain lesions and altered blood-brain barrier permeability in traumatic brain injury, ischemic stroke, cerebral tumors and subarachnoid hemorrhage. Early brain damage has been detected in patients with cirrhosis. We hypothesized that those insults could be detected by the PS-100 serum levels (normal values < 0.10 µg/L).

## Objectives

Hence, the aim of the present study was to assess PS-100 levels in cirrhotic patients, and compare them to PS-100 levels in non-cirrhotic patients followed up in the same department for initial workup of elevated liver enzymes (controls).

## Methods

We prospectively included a population of cirrhotic patients admitted for complication of cirrhosis in ICU and controls. Baseline data were recorded and levels of ammonemia and PS-100 determined at admission. Overt HE was diagnosed as a West-Haven score ranging between 2 and 4.

## Results

Between October 2013 and October 2014, 124 cirrhotic patients (mean age: 63 years, male gender: 73%, etiology of cirrhosis: alcoholic 51%, viral 25%, other 24%, Child-Pugh 11 [8-12], MELD score 21 [14-26]) and 79 non-cirrhotic patients (mean age: 63 years, male 59%, etiology of elevated liver enzymes: alcoholic 5%, viral 44%, NASH 38%, other 13%, mean Fibroscan: 5.3 kPa, mean FibroTest:0.15) were included. Among cirrhotic patients, 40 patients (33%) displayed HE at admission and 50 (41%) had an infection. Mean PS-100 level were 0,15 ± 0,01 for cirrhotic patients and 0,07 ± 0,01 for non-cirrhotic patients (p < 0.0001). In cirrhotic patients, PS-100 was correlated to AST (p = 0.0027), bilirubin (p = 0.0001), Child-Pugh score (p = 0.001) and MELD (p = 0.0004) and inversely correlated to albumin (p = 0.04), sodium (p = 0.01), PTT (p = 0.001), and FV (p = 0.011). PS-100 levels were not correlated to the presence of overt HE nor to infection. In multivariate analysis, levels of PS-100 were independently associated with MELD score (p = 0.0006), whereas overt HE was associated to hyperammonemia (p = 0.002) and the presence of infection (p = 0.008) but not to PS-100 levels.

## Conclusions

Patients with cirrhosis in ICU display neurological insult or blood-brain barrier dysfunction even in the absence of overt HE. Brain damage is more frequent in patients with advanced liver disease.

